# Knowledge Level and Associated Factors of Reproductive Health Issues among Secondary School Students in Woldia Town, Amhara, Ethiopia, 2019: A Cross-Sectional Study

**DOI:** 10.1155/2020/2515292

**Published:** 2020-10-22

**Authors:** Biruk Beletew Abate, Kalkidan Habtamu Gelaw, Hayelom Fentaw, Mekonen Ashagire, Tadesse Mekash

**Affiliations:** Department of Nursing, Faculty of Health Sciences, Woldia University, P.O. Box 400, Woldia, Ethiopia

## Abstract

**Background:**

Reproductive health (RH) is defined as a state of complete physical, mental, and social wellbeing and not merely the absence of disease or infirmity, in all matters related to the reproductive system and to its functions and process. Local evidence about adolescents' reproductive health knowledge level and associated factors are relevant to design age-appropriate interventions strategies. Therefore, the aim of this study was to assess the knowledge level on reproductive health issues among secondary school students in Woldia town.

**Objective:**

The main aim of this study was to assess the knowledge level and associated factors among adolescents in Woldia secondary schools, Amhara, Ethiopia, 2019.

**Methods:**

Institution-based descriptive cross-sectional study was conducted on 420 secondary school students in Woldia town from January to June 2019. Multistage sampling technique was employed. A self-administered, structured, and adapted questionnaire was used to collect the data. The data was entered by using EpiData version 4.2 and exported to SPSS version 24.0 for analysis. The samples were distributed proportionally based on probability proportional to size (PPS) allocation technique. Participants in each school have been selected by using systematic sampling technique after calculating sample interval (*K*) for each school. Bivariate and multivariable logistic regressions were carried out to assess the association between dependent and independent variables.

**Result:**

The prevalence of good knowledge was 204 (48.6%). Residence, educational level, handling of RHS providers, ever gone RHS institution and missed RHS service, had RHS in school, and stigma to utilize RHS were found to be significantly associated with the level of knowledge of respondents on reproductive health issues. *Conclusion and Recommendations*. The knowledge of respondents on reproductive health issues in the study area was found to be low. Hence, it is better to improve access to information to the secondary school students on RHS through trained health workers and accessible RHS.

## 1. Background

Reproductive health (RH) is defined as a state of complete physical, mental, and social wellbeing and not merely the absence of disease or infirmity, in all matters related to the reproductive system and to its functions and process. An adolescent is defined as a person aged between 10 and 19 years of age [1]. They account for 22% of the total population of Ethiopia [2].

Reproductive health is a universal concern, but is a special importance for women particularly during reproductive years [3]. It addresses the human sexuality and reproductive processes at all stages of life and implies that people are able to have a responsible, satisfying, and safe sex life and that they have the capability to reproduce and the freedom to decide if, when, and how often to do so. Youths and adolescents are characterized by unique physical, psychological, social, and emotional changes that put their life at high risk [2].

The term Youth Friendly Reproductive Health Service (YFRHS) refers to those services that are accessible, acceptable, and appropriate for the youth such as counseling, family planning, Voluntary Counseling and Testing (VCT), and treatment of Sexually Transmitted Infections (STI) [4]. The services are provided in line with the minimum health package and aims to increase acceptability and use of health services by young people [5]. Youth friendly services emphasize sexual and reproductive health counseling, contraceptive counseling and provision (including emergency contraception), STI prevention, HIV counseling and testing, treatment and care, prenatal and postpartum care, sexual abuse counseling, relationship counseling, and safe abortion and abortion-related services [6].

Currently, there is a low level of access to high-quality RH information and services, especially for adolescents [7]. In the past few years, the issues of RH have been increasingly perceived as a social problem; they have been emerging as a topic of increasing concern in both developed and developing countries [8]. Adolescents are not quite capable of understanding complex concepts. This makes them vulnerable to sexual exploitation and high-risk sexual behaviors and reproductive health problems [9].

Globally, 45 percent of all new HIV infections worldwide are occurring among young people aged 15 to 24 years; 500,000 young people are infected with an STI per day. Approximately 80 million women have unwanted pregnancies every year [10, 11]. Adolescent girls are considerably more likely to be infected than adolescent boys [12]. Many adolescents are less informed, less experienced, and less comfortable accessing health services for RH than do adults [13]. Moreover, there are unsafe abortions by young women aged 15–24, 45 percent, their fertility rate is high (116 percent), and the level of comprehensive knowledge of AIDS is 48 percent for young women aged 15–24 and 43 for young men aged 15–24 [4]. Young people in high schools and above are vulnerable and at risk to HIV infection due to various reasons (such as unprotected casual sex relationships and multiple sexual partners, lack of comprehensive knowledge about HIV/AIDS, sexual and reproductive health, lack of access to HIV services, sexual experimentation, early sexual debut and peer pressure, and other related factors) [14].

In Africa, 430,000 young people are infected with HIV per year; 2.6 million young people are living with HIV; and teenage pregnancy rates still remain high, and maternal mortality is among the leading causes of death for adolescent girls in this region [15].

The intergenerational gap between the generations of the youth and the adult is constantly being reinforced by cultural globalization. The comprehensive knowledge of HIV and other reproductive health (RH) problems is increasing; many young people do not have the information or means to protect themselves from these problems [16]. There are also problems related to SRH services which include accessibility, availability, and quality. Moreover, secondary school students indicated that health providers in most of the HEIs are not trained to respond to the needs of young persons [17]. The shortage of youth friendly health services and counseling poses significant challenges to address SRH issues, including HIV prevention.

In Ethiopia, educational institution-based RH services are often limited by restrictive policies, personnel shortages, lack of private areas for counseling, and poor links to resources outside the institutions. The problems of HIV/AIDS and other sexually transmitted infections (STIs) among youths indicating more efforts are needed toward sexual and reproductive health risk reduction interventions particularly in secondary school student. Hence, the main aim of the study was to assess the knowledge secondary school students on reproductive health issues in Woldia town.

## 2. Methods

### 2.1. Study Area and Period

This study was conducted in Woldia town from January to June 2019. Woldia town is located in the northeast Ethiopia Amhara regional state under the administration of North Wollo Zone, which is located at about 521 km from Addis Ababa in the west direction. This town has nine kebeles and four secondary schools. The study was conducted in all secondary schools, namely, Woldia secondary school, Millennium secondary school, Selam secondary school, and Gubo secondary school.

### 2.2. Study Design

The study design was institution-based descriptive cross-sectional study.

### 2.3. Source of Population

The source of population included all secondary school students in Woldia town.

### 2.4. Study Population

The study population involved all selected students of Woldia town secondary school.

### 2.5. Sample Size Determination

The sample size of the study was calculated using single proportion population formula as *n* = (Z^2^*α*/2 *∗* P  (1 − P)/*d*^2^), where *n* = sample size, *p* = proportion of sexual practice <18 years (taken as 53.3% according to reproductive health service utilization in Mizan-Tepi University students, Ethiopia, 2017 [3], *d* = maximum allowable error (margin of error) = 0.05, and *Z* = value of standard normal distribution at 95% confidence level (*z* *=* 1.96).

By adding 10% nonresponse rate, the total sample size will be 382 *∗* 10% = 38. Thus, the total sample size was *n* = 420.

### 2.6. Sampling Techniques and Procedure

We have used multistage stratified sampling technique. In the study area, there were a total of four secondary schools. Sections from each school were selected with simple random sampling method. Then, they were allocated proportionately. From each section, students were picked by systematic sampling method. The samples were distributed proportionally based on probability proportional to size (PPS). Participants in each school were selected by using systematic sampling technique after calculating sampling interval (*K*) for each school according to students' roll number in the class and the first student was selected by simple random method. *K* = *N*/*nf* = 3295/420 = 8. So, every 5 individuals were selected until the sample size was completed.

### 2.7. Variables

#### 2.7.1. Dependent Variables

The dependent variables included knowledge of reproductive health services, attitude towards reproductive health services, and practice of reproductive health services.

#### 2.7.2. Independent Variables

The independent variables included sociodemographic factors, health care factors, social factors, and behavioral factors.

### 2.8. Eligibility Criteria

#### 2.8.1. Inclusion Criteria

The inclusion criteria were Woldia town secondary school students who attend the class during data collection period and volunteers.

#### 2.8.2. Exclusion Criteria

- Those who were not available during data collection period
- Those who were physically and mentally not capable of being interviewed

### 2.9. Operational Definitions

#### 2.9.1. Good Knowledge

Those students who answer mean and above the mean score of knowledge questions were categorized as knowledgeable while those students answer below the mean score were categorized under poor knowledge.

#### 2.9.2. Good Attitude

Those students who answer mean and above the mean score of attitude questions were categorized as having good attitude while those students answer below the mean score were categorized as having poor attitude.

#### 2.9.3. Good Practice

Those students who answer mean and above the mean score of practice questions were categorized as having good practice while those students who answer below the mean score were categorized as having poor practice.

### 2.10. Data Collection Tool and Procedure

Data was collected by an adapted, pretested, structured, and interviewer-administered questionnaire. The questionnaire was first developed in English and translated to Amharic language by an expert. It was translated back to English by an independent translator to check for consistency. An interview with adolescent of the index students was conducted at their secondary school.

### 2.11. Data Quality Control

To assure data quality, the data collection tool was pretested. Data collectors and the supervisor were trained on the data collection techniques. In addition, the completeness, accuracy, and consistency of the collected data were checked on daily basis during the data collection time. The supervisors and principal investigators closely followed the data collection process. The data was entered on daily basis and missing data was identified. Incomplete data or questioners that miss the major content were not included in the study.

### 2.12. Data Analysis and Presentation

Data was entered by using EpiData version 4.2 and was exported to SPSS version 24 for analysis. Descriptive statistics like frequency, proportions, mean, and standard deviation were computed to describe the study variable in relation to the population and were presented by tables and graphs. Bivariate and multivariable logistic regressions were carried out to see associations between dependent and independent variables. Those variables that have *p* value ≤0.25 were taken to the multivariable logistic regression model to adjust for possible confounders. The strength of association was declared at *p* values <0.05. Finally, the result was compiled and presented using tables, graphs, and texts.

## 3. Result

All 420 respondents complete the questioner with the response rate of 100%. Among these, 204 (48.6%) were females. The majority of the respondents 238 (56.7%) were within the age group of 15–19 years. Most of respondents were unmarried 384 (91.4%) followed by having boy/girlfriend 231 (55.0%) (Table 1).

### 3.1. Knowledge Level of Respondents on Reproductive Health Issues

The prevalence of good knowledge was 204 (48.6%). About 334 (79.5%) respondents have awareness of pregnancy risk time. Three-fourths, 315 (75.0%), of respondents ever heard of VCT and the majority 322 (76.7%) of respondents ever heard about family planning method. One hundred sixteen (27.76%) respondents have got information about reproductive health services from their parents followed by 101 (24.16%) respondents who got from their teacher. The majority 292 (69.5%) of participants knows injectable as ways of contraception followed by condom 222 (52.9%). Regarding respondents' knowledge on STI, majority 362 (86.2%) of respondents had ever heard about STIs. Of those heard about STI, 58 (13.8%) participants have heard using radio/television. About 223 (53.1%), 146 (34.8%), and 123 (29.3%) of respondents know the most known types of STI: syphilis, gonorrhea, and chancroid, respectively. About 273 (65%), 163 (39%), and 270 (61%) of respondents know that STI manifested with genital discharge, genital ulcer, and pain during urination, respectively. Regarding knowledge about ways of HIV transmission, the majority 317 (75.5%) of respondents knows that unsafe sexual intercourse is one way of HIV/AIDS transmission. Regarding respondents' knowledge on ways to prevent unwanted pregnancies, the majority 258 (61.4%) of respondents know pills/injectable, 233 (55. 5%) and condom 86 (20.5%) as ways of prevention of unwanted pregnancy, respectively (Table 2).

### 3.2. Factors Associated with Knowledge Level of Respondents on Reproductive Health Issues

#### 3.2.1. Bivariate and Multivariate Analysis of Knowledge on RH Issues and Associated Factors

In bivariate logistic regression analysis residence, educational level, handling of RHS providers, ever gone RHS institution, had information about RHS, having girl/boyfriend, missed RHS service, had RHS in school, chew chat, and stigma to utilize RHS were associated with knowledge level of respondents on reproductive health issues. In multivariate logistic regression residence, educational level, handling of RHS providers, ever gone RHS institution, missed RHS service, had RHS in school, chew chat, and stigma to utilize RHS were found to be significantly associated with the level of knowledge of respondents on reproductive health issues. Accordingly, the odds of good knowledge on reproductive health issues among respondents from urban residence (AOR = 2.40, 95% CI (1.23, 2.36)) were higher compared to those respondents from rural residences. Respondents who are grade 10 were 1.46 times more likely to have good level knowledge on RH issues compared to grade 9 respondents. The odds of good knowledge on reproductive health issues among respondents who get good handling from RHS providers (AOR = 2.28, 95% CI (1.15, 2.54)) were higher compared to those who get bad handling from RHS providers. Respondents who ever gone to RHS and did not miss the service were 1.25 times more likely to have good knowledge of RH issues compared to those who missed the service. The odds of good knowledge on reproductive health issues among respondents who have RHS in their school (AOR = 2.06, 95% CI (1.68, 7.66)) were higher compared to those who have not RHS in their school. Moreover respondents who live in a community with stigma to utilize RHS were 37% less likely to have good knowledge on RH issues compared to those who live in a community with no stigma to utilize RHS (Table 3).

## 4. Discussion

This institution-based descriptive cross-sectional study was conducted on 420 study participants to assess the magnitude and associated factors of knowledge on reproductive health services among secondary school students in Woldia town. Accordingly, the overall prevalence of good knowledge was 48.6%. This finding is consistent with two studies conducted in Nigeria [18, 19]. This might be due to the similarity of study subjects in both Nigerian studies, and our studies are conducted on adolescents. The magnitude is lower than that of the study conducted in Gondar University [20] and Wolaita Sodo University [21]. The possible reason could be the difference in the study population that the studies from Gondar and Wolaita Sodo University are conducted on university students who have better exposure on RH issues compared with our study subjects who are secondary school students having less information exposure. The high proportion health science students in their study may also be the possible reason. Students who come from urban areas are more likely to have good level of knowledge about HR issues than those who come from rural areas. This finding is in line with a study conducted in Wolaita Sodo University [21] and in Gondar University [20]. As evidenced from the Ethiopian demographic and health survey, people from rural areas are less likely to read a newspaper, listen to the radio, or watch television; therefore, their knowledge about HR issues can be negatively affected [22]. Respondents who are grade 10 were more likely to have good level of knowledge on RH issues compared to grade 9 respondents. This finding is supported by another studies in Ethiopia [22, 23]. This might be due to the fact that as the level of education increases, the information exposure about RH issues and age maturity will improve the knowledge level of students. The odds of good knowledge on reproductive health issues among respondents who get good handling from RHS providers were higher compared to those who get bad handling from RHS providers. This might be due the fact that good handling of RH service providers would help the participants to have positive attitude towards SRH services and they ask for information regarding RH issues and they will listen to and apply the counseling given by the providers [23]. The odds of good knowledge on reproductive health issues among respondents who have RHS in their school were higher compared to those who have not RHS in their school. This finding is supported by the study conducted in northeast Ethiopia [23]. The possible justification for this might be those service providers in school will give counseling on RH issues in addition to giving treatment. In these settings, students are more likely to get education about SRHRs while they visit the clinic for other RH services which boosts their knowledge. The odds of good level of knowledge on RH issues are lower among those adolescents who have stigma compared to those who have no stigma have higher risk of low level to poor RH utilization than their counterparts. This result is similar with Nigeria and Nepal [24, 25]. This might be due to the fact that those who were stigmatized lack proper information on safe sex as well as they cannot communicate their problems with each other in fear of social stigma and less likely to get the opportunity to RH service and counseling.

### 4.1. Strength and Limitation

The study used adapted standardized questionnaires which increase its validity. Adequate sample size was employed with a high response rate. The investigators made a lot of effort to maintain the quality of the data, mainly through a pretest, frequent field supervisions, and training of data collectors. However, the study is not free from error. It may introduce social desirability and recall bias. In addition, the study was not supplemented with any qualitative data. As being cross-sectional, this work has not shown cause-effect relationships.

## 5. Conclusion

The level of knowledge of respondents on reproductive health issues in the study area was low. Residence, educational level, handling of RHS providers, ever gone RHS institution, missed RHS service, had RHS in school, chew chat, and stigma to utilize RHS were found to be significantly associated with the level of knowledge of respondents on reproductive health issues. Hence, strengthening women's education and establishing RH service and RH issues-related education shall be available among all faculties giving emphasis to students from rural areas. Information about reproductive health issues should be provided to adolescents through medical schools' curricula.

## Figures and Tables

**Table 1:** Sociodemographic characteristics of secondary school students in Woldia town, Amhara, Ethiopia, 2019.

Characteristics	Category	Frequency	Percent
Age	10–15	50	11.9
	15–19	238	56.7
	20–24	132	31.4

Sex	Male	216	51.4
	Female	204	48.6

Marital status	Unmarried	384	91.4
	Married	21	5.0
	Divorced	13	3.1
	Widowed	2	.5

Residence	Urban	304	72.4
	Rural	116	27.6

Father's occupation	Farmer	159	159
	Civil servant	131	131
	Merchant	72	72
	Driver	34	34
	Daily laborer	4	4
	Others	20	20

Wealth index	High	108	25.7
	Medium	245	58.3
	Low	67	16.0

Ethnicity	Amhara	398	94.8
	Tigri	17	4.0
	Others	5	1.2

Religion	Muslim	122	29.0
	Orthodox	278	66.2
	Protestant	14	3.3
	Others	6	1.4

Educational level	Grade 9	237	56.4
	Grade 10	183	43.6

Presence of RHS in school	Yes	286	68.1
	No	134	31.9

Handling of RHS providers	Good	191	46
	Moderate	176	41.9
	Bad	51	12.1

Missed RHS required	Yes	262	62.4
	No	158	37.6

Reason for not getting RH	Lack of money	156	37.1
	Neighbors felt ashamed	147	35.0
	Service providers refused	77	18.3
	Clinic was closed	40	9.5

Ever heard of YFRHS	Yes	261	62.1
	No	159	37.9

Parents influence not to use RHS	Yes	270	64.3
	No	150	35.7

Stigma attached to utilizing RHS	Yes	268	63.8
	No	152	36.2

Cultural and religious influence	Yes	248	59.0
	No	172	41.0

Have girl/boyfriend	Yes	231	55.0
	No	189	45.0

Had sexual intercourse	Yes	150	35.7
	No	270	64.3
	No	248	59.0

Chew chat	Yes	103	24.5
	No	317	75.5

Drink alcohol	Yes	122	29.0
	No	298	71.0

**Table 2:** Knowledge of RHS among secondary school students in Woldia, North Wollo Zone, Ethiopia, 2019.

Variables	Category	Frequency	Percent
Aware ness of pregnancy risk time	Yes	334	79.5
	No	86	20.5

RH information	Yes	298	71
	No	122	29

Ever heard about family planning method	Yes	322	76.7
	No	98	23.3

Ever heard of VCT	Yes	315	75.0
	No	105	25.0

Source of information about RH	Parents	116	27.76
	Peer	99	23.76
	Teacher	101	24.16
	Notice board	71	17.06
	Mass media	33	7.26

*Ways of preventing unwanted pregnancy*			

Oral contraceptive pills	Yes	231	55.0
	No	189	45.0

Condom	Yes	222	52.9
	No	198	47.1

Injectable	Yes	292	69.5
	No	128	30.5

IUCD	Yes	77	18.3
	No	343	81.7

Sterilization	Yes	51	12.1
	No	369	87.9

Abstain	Yes	99	23.6
	No	321	76.4

Withdrawal	Yes	34	8.1
	No	386	91.9

Safe period/abstain	Yes	61	14.5
	No	359	85.5

Ever heard of STI	Yes	362	86.2
	No	58	13.8

*Most known types of STI*			

Gonorrhea	Yes	146	34.8
	No	274	65.2

Syphilis	Yes	223	53.1
	No	197	46.9

Chancroid	Yes	123	29.3
	No	297	70.7

LGV	Yes	75	17.9
	No	345	82.1

HIV/AIDS	Yes	325	77.4
	No	95	22.6

*Symptoms of STI*			

Genital discharge	Yes	273	65
	No	147	35

Genital ulcer	Yes	163	39
	No	257	61

Pain during urination	Yes	270	61
	No	150	39

*Ways of HIV/AIDS transmission*			

Unsafe sexual intercourse	Yes	317	75.5
	No	103	24.5

Blood transfusion	Yes	228	54.3
	No	192	45.7

Sharing of needle and syringes	Yes	290	69.0
	No	130	31.0

During pregnancy and child birth	Yes	149	35.5
	No	271	64.5

Through breast milk	Yes	131	31.2
	No	289	68.8

Through mosquito and other insects' bite	No	324	77.1
	Yes	96	22.9

Hand shaking or sharing foods	No	330	78.6
	Yes	90	21.4

*Ways of preventing unwanted pregnancy*			

Abstain	Yes	242	57.6
	No	178	42.4

Condom	Yes	233	55.5
	No	187	44.5

Pills/injectable	Yes	258	61.4
	No	162	38.6

Abstain	Yes	242	57.6
	No	178	42.4

**Table 3:** Factors associated with knowledge of RH issues among secondary school students in Woldia, North Wollo Zone, Ethiopia, 2019.

Exposure variable	Responses	Knowledge level		COR with 95% CI	AOR 95% CI	*p*value
		Good knowledge 204 (48.6%)	Poor knowledge 216 (51.4)			
Residence	Urban	137 (32.6)	167 (39.8)	3.67 (1.08, 2.57)	**2.40 (1.23, 2.362)** ^*∗*^	**0.024**
	Rural	67 (16.0)	49 (11.7)	1	1	—

Wealth index	High	47 (11.2)	61 (14.5)	1	1	—
	Medium	125 (29.8)	120 (28.6)	0.74 (0.47, 1.17)	0.96 (0.58, 1.580)	0.868
	Low	32 (7.6)	35 (8.3)	0.84 (0.46, 1.55)	1.18 (0.60, 2.32)	0.632

Educational level	Grade 9	102 (24.3)	135 (32.1)	1	1	—
	Grade 10	102 (24.3)	81 (19.3)	1.67 (1.13, 2.46)	**1.46 (1.16, 2.21)** ^*∗*^	**0.036**

Had information about SRHS	Yes	152 (36.2)	182 (43.3)	1.83 (1.13, 2 .97)	1.56 (0.92, 2.63)	.098
	No	52 (12.4)	34 (8.1)	1	1	—

Handling of RHS providers	Good	84 (20.0)	108 (25.8)	2.45 (0.78, 2.69)	**2.28 (1.15, 2.54)** ^*∗*^	**0.047**
	Moderate	93 (22.2)	83 (19.8)	1.00 (0.54, 1.88)	0.98 (0.50, 1.91)	0.949
	Bad	27 (6.4)	24 (5.7)	1	1	0.323

Ever gone RHS and missed the service	Yes	120 (28.6)	142 (33.8)	1	1	—
	No	84 (20.0)	749 (17.6)	1.34 (90, 2.00)	**1.25 (1.10, 5.94)** ^*∗*^	**0.023**

Had RHS in school	Yes	121 (28.8)	143 (34.0)	2.34 (0.90, 2.00)	**2.06 (1.68, 7.66)** ^*∗*^	**0.011**
	No	83 (19.8)	73 (17.4)	1	1	—

Is there stigma to utilize RHS?	Yes	138 (32.9)	130 (31.0)	0.73 (0.48, 1.08)	**0.63 (0.41, 0.98)** ^*∗*^	**0.041**
	No	66 (15.7)	86 (20.5)	1	1	—

Do you have girl/boyfriend?	Yes	106 (25.2)	125 (29.8)	1.27 (0.86, 1.87)	1.14 (0.74, 1.77)	0.556
	No	98 (23.3)	91 (21.7)	1	—	—

Did you ever had sexual intercourse?	Yes	67 (16.0)	83 (19.8)	1.28 (0.86, 1.91)	0.98 (0.61, 1.580)	0.935
	No	137 (32.6)	133 (31.7)	1	1	—

Did your friends encourage you to use RHS?	Yes	77 (18.3)	95 (22.6)	10 (0.88, 1.91)	0.78 (0.47, 1.28)	0.320
	No	127 (30.2)	121 (28.8)	1	1	—

Did you chew chat?	Yes	36 (8.6)	67 (16.0)	2.10 (1.32, 3.33)	**1.85 (1.01, 3.38)** ^*∗*^	**0.047**
	No	168 (40.0)	149 (35.5)	1	1	—

Did you drink alcohol?	Yes	47 (11.2)	75 (17.9)	1.78 (1.16, 2.73)	1.31 (0.75, 2.30)	0.345
	No	157 (37.4)	141 (33.6)	1	1	—

*Note.* ^*∗*^ *p* value < 0.05, CI = confidence interval, COR = crude odds ratio, and AOR = adjusted odds ratio.

## Data Availability

The data used to support the findings of this study are available from the corresponding author upon request.
